# Expanded Potential of the Polyamine Analogue SBP-101 (Diethyl Dihydroxyhomospermine) as a Modulator of Polyamine Metabolism and Cancer Therapeutic

**DOI:** 10.3390/ijms23126798

**Published:** 2022-06-18

**Authors:** Cassandra E. Holbert, Jackson R. Foley, Tracy Murray Stewart, Robert A. Casero

**Affiliations:** Sidney Kimmel Comprehensive Cancer Center, Baltimore, MD 21231, USA; cholber2@jhmi.edu (C.E.H.); jfoley13@jhmi.edu (J.R.F.); tmurray2@jhmi.edu (T.M.S.)

**Keywords:** polyamine, polyamine analogue, polyamine metabolism, pancreatic cancer, ovarian cancer, cancer therapy, drug development

## Abstract

Naturally occurring polyamines are absolutely required for cellular growth and proliferation. Many neoplastic cells are reliant on elevated polyamine levels and maintain these levels through dysregulated polyamine metabolism. The modulation of polyamine metabolism is thus a promising avenue for cancer therapeutics and has been attempted with numerous molecules, including enzyme inhibitors and polyamine analogues. SBP-101 (diethyl dihydroxyhomospermine) is a spermine analogue that has shown efficacy in slowing pancreatic tumor progression both in vitro and in vivo; however, the mechanisms underlying these effects remain unclear. We determined the effects of the SBP-101 treatment on a variety of cancer cell types in vitro, including lung, pancreatic, and ovarian. We evaluated the activity of enzymes involved in polyamine metabolism and the effect on intracellular polyamine pools following the SBP-101 treatment. The SBP-101 treatment produced a modest but variable increase in polyamine catabolism; however, a robust downregulation of the activity of the biosynthetic enzyme, ornithine decarboxylase (ODC), was seen across all of the cell types studied and indicates that SBP-101 likely exerts its effect predominately through the downregulation of ODC, with a minor upregulation of catabolism. Our in vitro work indicated that SBP-101 was most toxic in the tested ovarian cell lines. Therefore, we evaluated the efficacy of SBP-101 as a monotherapy in the immunosuppressive VDID8^+^ murine ovarian model. Mice treated with SBP-101 demonstrated a delay in tumor progression, a decrease in the overall tumor burden, and a marked increase in median survival.

## 1. Introduction

The naturally occurring mammalian polyamines, spermine, spermidine, and putrescine, are small, polycationic alkylamines that are positively charged at a physiological pH, allowing for interactions with negatively charged macromolecules, including DNA, RNA, and certain proteins [1,2]. Due to these interactions, polyamines are involved in numerous critical cell processes and are essential for cellular growth, proliferation, and survival. Polyamines exist naturally at millimolar concentrations in eukaryotic cells, and while unbound concentrations are thought to be much lower, the maintenance of polyamine homeostasis is absolutely critical to cellular viability. Due to their continual proliferation, cancer cells of all types exhibit elevated intracellular polyamine pools that are maintained through a dysregulated polyamine metabolism.

The dysregulated polyamine metabolism in tumors can involve the upregulation of polyamine biosynthesis, the downregulation of polyamine catabolism, an increase in the uptake of extracellular polyamines [3,4,5], or any combination of the three. Many oncogenes, including *myc, ras*, and *jun*, are directly involved in the maintenance of elevated polyamine levels [6,7,8]. Of note, the upregulation of ornithine decarboxylase (ODC), a rate-limiting enzyme in polyamine biosynthesis that is a direct target of MYC, has been correlated with increased polyamine pools in nearly every type of cancer [6]. Due to its direct link to oncogenes and the dysregulation by neoplastic cells, polyamine metabolism is a long-standing target for potential cancer therapeutics [9]. 

As polyamine concentrations are tightly regulated, a promising therapeutic strategy for exploiting their self-regulation is through the use of polyamine analogues. A major class of these analogues involves the alkylation of the primary amine groups of spermine [10]. These compounds traditionally compete with native polyamines for uptake and, upon intracellular accumulation, stimulate the catabolism of higher-order polyamines and reduce polyamine biosynthesis [11]. In order to be effective, polyamine analogues must compete with natural polyamines for uptake into the cell and induce a negative feedback inhibition sufficient to decrease the intracellular pools of natural polyamines. Importantly, therapeutically successful polyamine analogues must be sufficiently dissimilar to the natural polyamines so as not to replace the cellular functions of the polyamines. 

**Figure 1:**
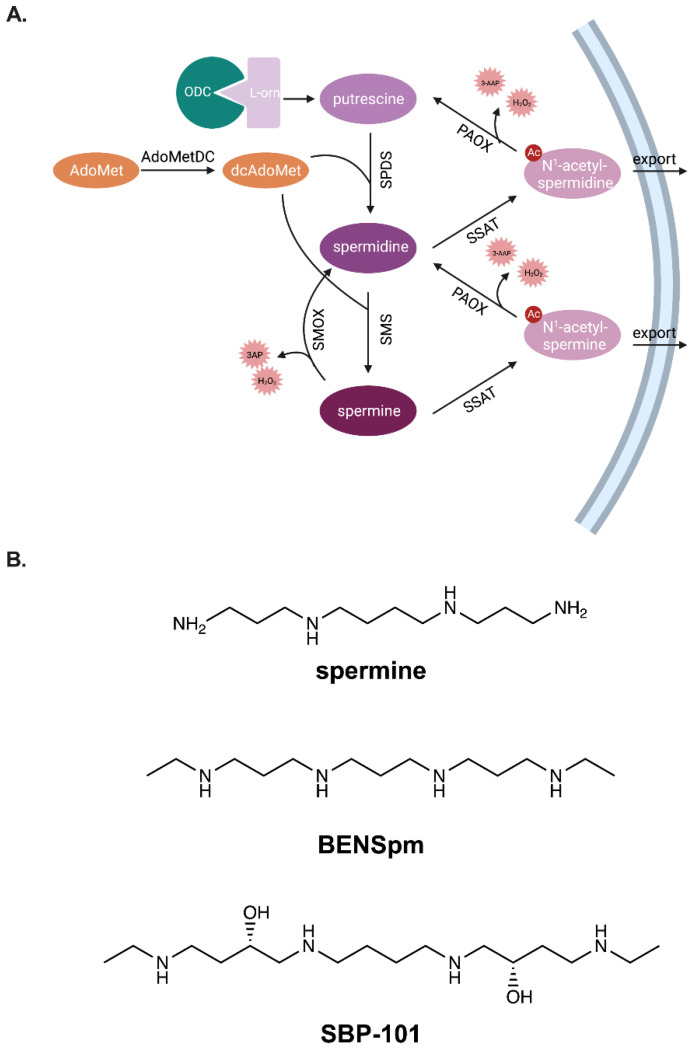
**Polyamine metabolism is regulated through coordinated biosynthesis, catabolism, and transport.** Ornithine decarboxylase (ODC) is a rate-limiting enzyme in polyamine biosynthesis (**A**). It catalyzes the formation of putrescine from L-ornithine. Spermidine synthase (SPDS) catalyzes the formation of spermidine from putrescine, and spermine synthase (SMS) assists in producing spermine from spermidine. Both reactions require decarboxylated adenosylmethionine produced by S-adenosylmethionine decarboxylase (AdoMetDC), the second rate-limiting enzyme in polyamine biosynthesis. Spermine oxidase (SMOX) directly catabolizes spermine to spermidine and produces the toxic byproducts, 3-aminopropanal and hydrogen peroxide. Alternatively, spermine can be acetylated by spermidine/spermine *N*^1^-acetltransferase (SSAT) to form *N*^1^-acetyl-spermine, which can be exported from the cell or further oxidized by polyamine oxidase (PAOX) to form spermidine. Spermidine can be catabolized to putrescine in a similar manner, forming *N*^1^-acetyl-spermidine as an intermediary. Analogues of the polyamine spermine compete for uptake into cells, and their accumulation influences polyamine metabolism (**B**). The well-characterized polyamine analogue, *N*^1^,*N*^11^-bisethylnorspermine, influences ODC, AdoMetDC, SSAT, and SMOX to affect polyamine metabolism. The effects of the spermine analogue, SBP-101, on polyamine metabolism are addressed in this manuscript.

The spermine analogue, N^1^,N^11^-bis(ethyl)norspermine (BENSpm, also known as DENSpm), is a symmetrically substituted spermine analogue with N-terminal ethyl groups [12]. BENSpm is biochemically well-characterized and known to influence enzymes involved in polyamine metabolism (Figure 1) [13,14,15,16]. While BENSpm causes the remarkable cytotoxicity of cancer cells in vitro, notable off-target effects and toxicities from BENSpm in clinical trials resulted in a slowed interest in first-generation polyamine analogues as cancer therapeutics [17,18]. While it is likely that utilizing lower doses of first-generation analogues in the clinic would have resulted in much lower toxicities, the field has progressed to developing new derivatives of the original polyamine analogues, such as PG-11047 and SBP-101. PG-11047 is a second-generation analogue with a conformationally restricting central *cis* double bond aimed at reducing the off-target effects of BENSpm [18,19,20]. While preclinical animal models have shown PG-11047 to delay tumor progression and extend survival, the clinical dosing schedules used to date have only managed to maintain stable disease [19,20,21,22]. 

SBP-101 (diethyl dihydroxyhomospermine) is a spermine analogue with many structural similarities to BENSpm [23]. In a Phase 1A/1B clinical study, the combination of existing FDA-approved standard chemotherapy and SBP-101 has shown the inhibition of tumor growth in pancreatic cancer patients without exacerbating bone marrow suppression or peripheral neuropathy [24]. The multi-center ASPIRE Phase 2/3 clinical study combining SBP-101 with nab-paclitaxel and gemcitabine in metastatic pancreatic ductal adenocarcinoma is currently enrolling patients (NCT05254171) [25]. Though SBP-101 has been introduced into the clinic, its full influence on polyamine metabolism and the mechanisms of action remain to be determined. Here, we investigate the impact of the SBP-101 treatment on polyamine metabolism and polyamine content, as well as explore the potential for SBP-101 as a treatment in cancers other than pancreatic. 

## 2. Results

### 2.1. SBP-101 Treatment Reduces Cell Viability in Lung, Pancreatic, and Ovarian Cancers In Vitro

**Figure 2:**
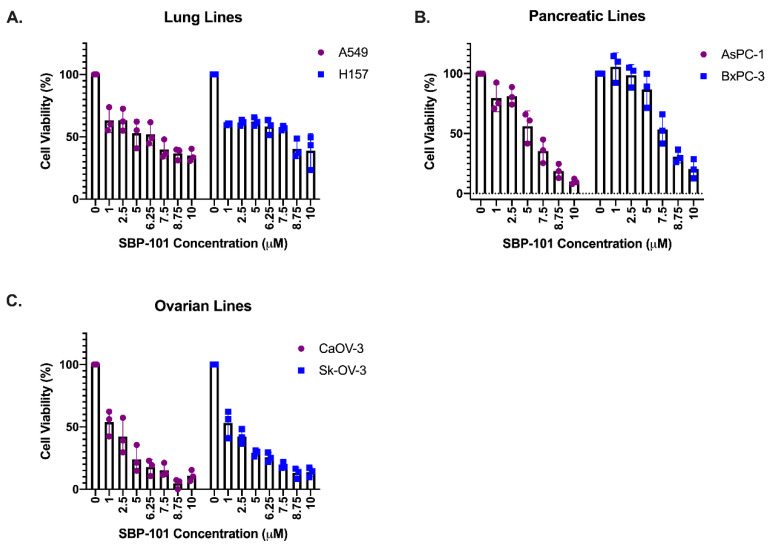
**SBP-101 treatment reduces cancer cell viability in vitro.** Lung, pancreatic, and ovarian adenocarcinoma lines were treated with SBP-101 concentrations ranging from 1 to 10μΜ. Cells were collected, and the number of viable cells (determined by trypan blue exclusion) was counted following treatment. SBP-101 treatment reduced the cellular viability in all treated lung (**A**), pancreatic (**B**), and ovarian (**C**) cancer cell lines. IC_50_ values are listed in Table 1.

Lung adenocarcinoma lines, A549 and H157, and pancreatic adenocarcinoma lines, AsPc-1 and BxPC-3, were treated for 96 h with concentrations of SBP-101 ranging from 1 μM to 10 μM. Due to their longer doubling time, ovarian adenocarcinoma lines (CaOV-3 and Sk-OV-3) were treated with SBP-101 over 120 h. All of the cell lines exhibited at least a 50% reduction in viability within the tested concentration range. The lung and pancreatic cancer cell lines showed variable responses: the lung lines A549 and H157 exhibited IC_50_ values of <7.0 μM (Figure 2A). AsPc-1 pancreatic cancer cells had an IC_50_ of <5.0 μΜ in response to SBP-101, while BxPC-3 cells showed the least viability reduction of all tested cell lines, with an IC_50_ of ~9.0 μM (Figure 2B). The CaOV-3 and Sk-OV-3 ovarian lines showed the greatest reduction in viability, with IC_50_ values of <2.0 μM (Figure 2C). The IC_50_s values for each cell line are listed in Table 1.

### 2.2. SBP-101 Treatment Reduces Intracellular Polyamine Content 

**Figure 3:**
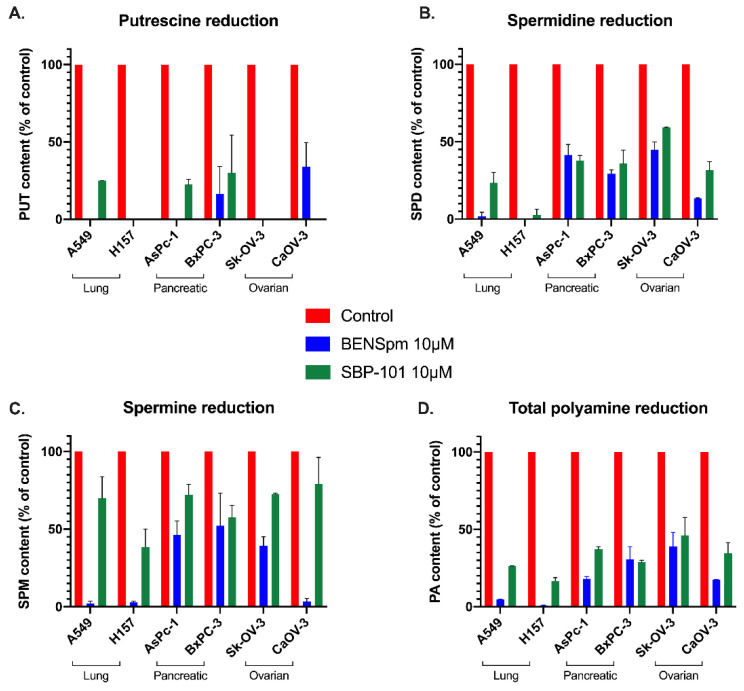
**SBP-101 treatment results in a decrease in intracellular polyamine content.** Lung, pancreatic, and ovarian adenocarcinoma lines were treated with 10 μM of either BENSpm or SBP-101 for 24 h. Cells were then collected and analyzed by HPLC for intracellular polyamine content. Both BENSpm and SBP-101 reduced intracellular putrescine levels to under 25% of original levels in all cell lines, except BxPC-3 (**A**). Both analogues reduced spermidine content by at least 50% in all treated cell lines, with lung lines showing the most reduction and pancreatic lines showing the least (**B**). BENSpm treatment decreased spermine levels in all cell lines but with varied intensity. While SBP-101 showed little reduction in spermine levels across the tested cell lines, overall polyamine levels decreased with treatment (**C**,**D**).

The spermine analogue BENSpm is biochemically well-characterized for its ability to influence polyamine metabolism and deplete intracellular polyamine pools. BENSpm has been shown to decrease polyamine pools by downregulating both rate-limiting biosynthetic enzymes, ODC and S-adenosylmethionine decarboxylase (AdoMetDC), as well as inducing spermidine/spermine *N*^1^-acetyltransferase (SSAT) and spermine oxidase (SMOX) [13,14,15,16]. Lung, pancreatic, and ovarian adenocarcinoma cell lines were treated for 24 h with 10 μM of either BENSpm or SBP-101 to compare how these molecules influence the polyamine pools. The treatment with SBP-101 resulted in at least a 75% reduction in putrescine levels in all of the tested cell lines, except BxPC-3 (Figure 3A). The BENSpm treatment produced a similar response with all of the cell lines (except BxPC-3), reducing the putrescine levels to below detectable amounts. The SBP-101 treatment produced a reduction in spermidine levels in all of the treated cell lines (Figure 3B). AsPc-1, BxPC-3, and CaOV-3 showed an approximately 65% reduction in the spermidine levels, while Sk-OV-3, A549, and H157 showed reductions ranging between 85% and 100%. BENSpm similarly reduced the spermidine levels in all of the cell lines. The spermine levels showed a limited response to the SBP-101 treatment, with only two cell lines showing a reduction of spermine levels near 50% (Figure 3C). The BENSpm treatment resulted in a varying reduction in the spermine levels in all of the cell lines, ranging from 55–95%. The SBP-101 treatment reduced the overall polyamine content by at least 50% in all of the test cell lines (Figure 3D). Both analogues, BENSpm and SBP-101, were successfully taken up by the cells (Table 2), and while the accumulation varies slightly between the cell lines, the concentration of the intracellular analogue does not directly correlate to the cell line response. 

### 2.3. Polyamine Metabolic Enzyme Activity Is Influenced by SBP-101 Treatment

**Figure 4:**
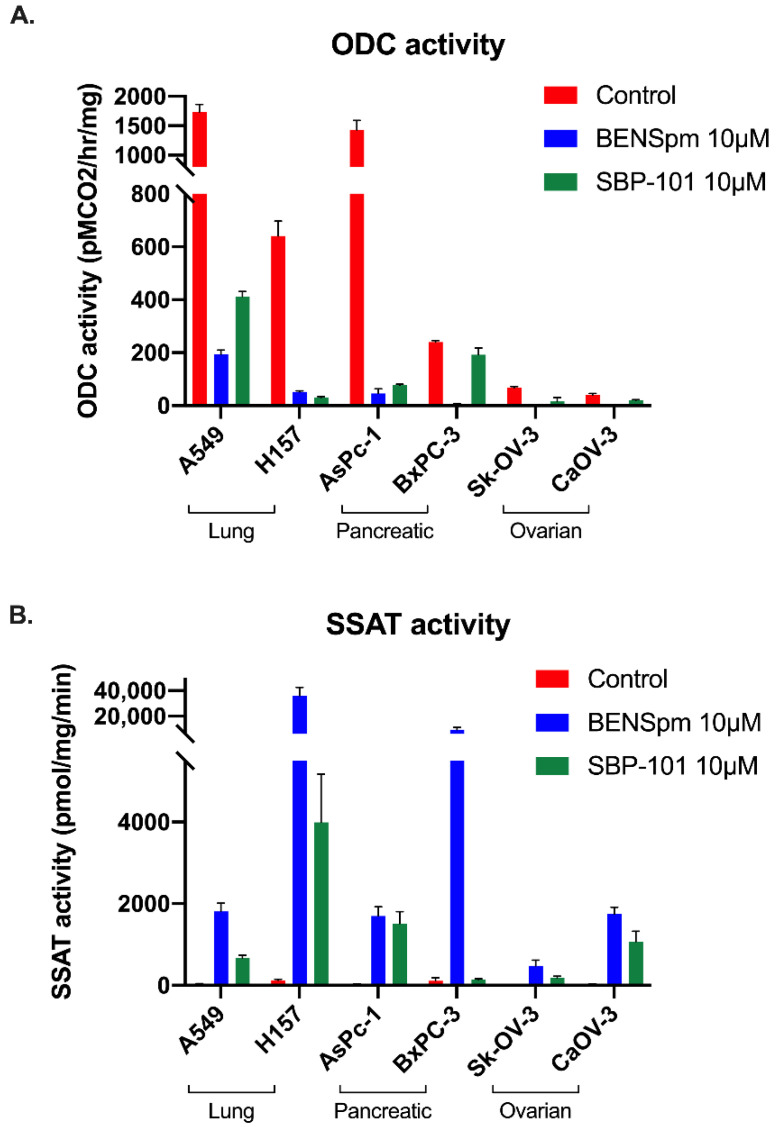
**Polyamine metabolism is influenced by SBP-101 treatment through ODC inhibition and modest SSAT upregulation.** Following treatment with 10 μM of BENSpm or SBP-101 for 24 h, lung, pancreatic, and ovarian adenocarcinoma cells were collected, and lysates were tested for ODC and SSAT enzyme activity. Both BENSpm and SBP-101 substantially reduced the activity of the biosynthetic enzyme ODC (**A**). BENSpm treatment resulted in the induction of the catabolic enzyme SSAT in all treated cell lines. SBP-101 also upregulated SSAT activity, albeit to lower levels than BENSpm treatment (**B**).

Using lysates from cells treated for 24 h with 10 μΜ of either ΒΕΝSpm or SBP-101, the activity of major polyamine metabolic enzymes was determined. As expected, the BENSpm treatment reduced the ODC activity in all of the cell lines. The SBP-101 treatment reduced the ODC activity in all of the cell lines tested, except BxPC-3. In both of the ovarian adenocarcinoma lines, the SBP-101 treatment caused a reduction in the ODC activity to nearly non-detectable levels (Figure 4A). All of the tested cell lines exhibited a significant upregulation of the catabolic enzyme SSAT following the BENSpm treatment (Figure 4B). The SBP-101 treatment, however, resulted in a variable and more modest upregulation of SSAT. The AsPC-1 and H157 cell lines robustly upregulated SSAT in response to SBP-101, while the A549 and CaOV-3 cell lines exhibited a less extensive upregulation. The BxPC-3 and Sk-OV-3 cells showed little to no upregulation of SSAT in response to the SBP-101 treatment. The preliminary results indicate that SBP-101 does not influence AdoMetDC or SMOX activities (data not shown). 

### 2.4. SBP-101 Treatment Prolongs Survival and Reduces Tumor Burden in the VDID8^+^ Ovarian Murine Model

**Figure 5:**
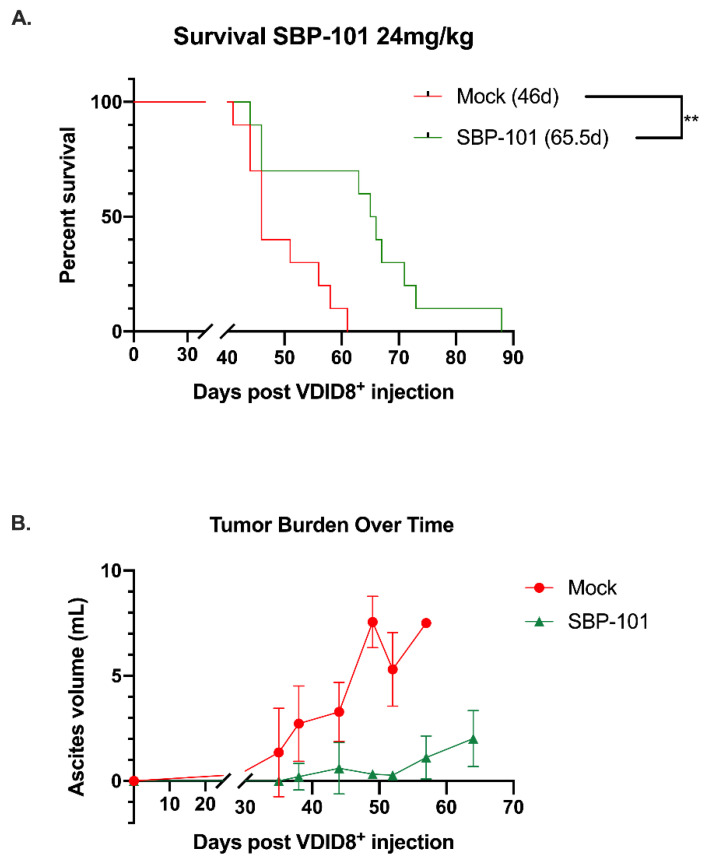
SBP-101-treated VDID8^+^ mice (*n* = 10) show a delay in tumor progression and a decrease in overall tumor burden that is correlated with an increase in median survival. Female C57Bl/6 mice were injected with 250,000 VDID8^+^ mouse ovarian surface epithelial cells and subsequently treated with 24 mg/kg SBP-101 (3× per week, alternating weeks, three cycles). The median survival for treated mice was 65.5 days, while the median survival for untreated mice was 46 days (**A**). Untreated mice produced measurable ascites prior to treated mice, and the overall volume of ascites in untreated mice was larger (**B**). Using ascites as a measure of tumor burden, mice treated with SBP-101 were delayed in tumor formation and exhibited an overall decrease in tumor burden.

Following an I.P. implantation with VEGF-β-Defensin ID8 (VDID8^+^) syngeneic mouse ovarian surface epithelial cells, C57BL/6 mice were treated with 24 mg/kg SBP-101 three times per week on alternating weeks. Due to the weight loss observed in this high-dose group, the treatment with SBP-101 was discontinued after three cycles (five weeks) (Supplementary Figure S1A). These mice continued to be followed for weight change and survival, and ascites and tumor samples were processed for further analysis. An additional experiment was then completed using either a lower dose of 6 mg/kg, 3× per week on alternating weeks or 24 mg/kg once per week on alternating weeks. Neither dose on this schedule showed any weight-related toxicities (Supplementary Figure S1B). Both doses of SBP-101 prolonged the survival of the mice when given 3× per week, with the original high dose (24 mg/kg) yielding a 65-day median survival and the low dose (6 mg/kg) giving a 62-day median survival (Figure 5A, Supplementary Figure S1C). The median survival of either dose was significantly longer than that of both groups of the control mice (46 days), with *p*-values of 0.0022 (high dose) and 0.0067 (low dose). The mice treated with 24 mg/kg once each alternating week had a median survival of 55 days, which was not statistically significant, with a *p*-value of 0.0908 (Supplementary Figure S1C). 

**Figure 6:**
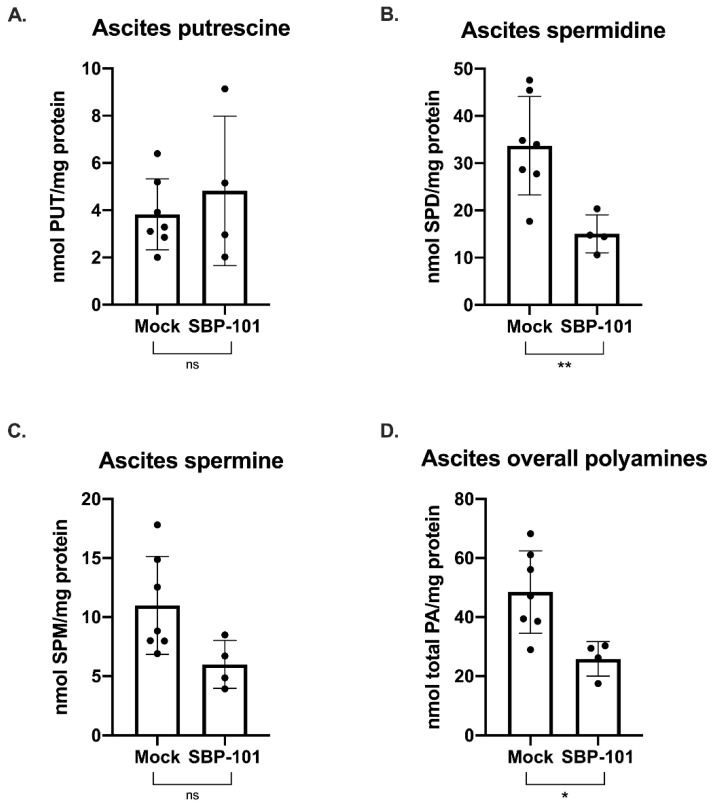
**SBP-101 results in decreased polyamine content in the cells of the ascites fluid of treated mice.** Ascites was drained from a subset of untreated mice and mice treated with 24 mg/kg SBP-101 (3× alternating weeks) 52 days post-VDID8^+^ injection. Red blood cells were lysed from drained ascites fluid, and the remaining cells were analyzed for polyamine content by HPLC. There was no significant difference in putrescine levels between treated and untreated mice (**A**). Spermidine levels in the ascites cells were significantly decreased in SBP-101-treated mice with a *p*-value of 0.008 (**B**). Spermine levels were also lower in the ascites cells of treated mice, though the observed decrease was not statistically significant (*p*-value = 0.0534) (**C**). Overall polyamine levels were significantly lower in SBP-101-treated mice (*p*-value = 0.014) (**D**). Significance symbols are as follows: ns = not significant (*p*-value > 0.05); * = *p*-value < 0.05; ** = *p*-value < 0.01.

In this mouse model, the ascites volume can be used as an indicator of tumor burden. Ascites fluid was collected and processed from mock and SBP-101-treated mice throughout the original study (24 mg/kg SBP-101 3× per week, alternating weeks). The ascites was collected from mice showing more than a 20% increase in body weight (typically once or twice per week following the original ascites formation). The ascites for a polyamine analysis were collected 52 days post-VDID8^+^ injection, while there was a sufficient number of ascites-producing animals from both arms of the experiment. The SBP-101-treated mice showed a significant delay in the ascites formation and an overall decrease in tumor burden (Figure 5Β). A subset of the ascites fluid was analyzed by HPLC to determine changes in the polyamine levels with the SBP-101 treatment. There were reductions in spermidine, spermine, and the overall polyamine levels in the ascites of mice treated with SBP-101 (*p*-values: 0.008, 0.0534, and 0.014, respectively) (Figure 6). The SBP-101 levels were measured by HPLC in the kidneys and livers harvested from a subset of these mice during necropsy. SBP-101 accumulated in both the liver and the kidneys and influenced the polyamine levels in these organs. (Supplementary Table S1). 

## 3. Discussion

Cancer cells depend on an aberrant polyamine metabolism to maintain the elevated polyamine levels required for continual self-renewal [5,26]. Many attempts have been made at enzymatic inhibitors; however, the difficulties associated with monotherapeutic enzyme inhibition have led to an expanded interest in compounds that further regulate polyamine metabolism. Polyamine analogues are molecules structurally similar to the native polyamines that are recognized and transported by the polyamine transport system but are structurally dissimilar enough that they are unable to functionally replace the natural polyamines [27]. The accumulation of polyamine analogues can influence polyamine metabolism through the regulation of the associated enzymes. 

SBP-101 is a spermine analogue that has been shown to slow pancreatic tumor growth in vitro and in vivo and is currently enrolling in a Phase 2/3 clinical trial (NCT05254171) [25]. Here, we evaluated the direct influence of SBP-101 on the polyamine content and metabolism, which have not previously been determined. Our in vitro studies determined that SBP-101 reduces cellular viability across a broad range of cancer cell types, with an exceptionally strong reduction in ovarian adenocarcinoma viability. The reduction in cellular viability corresponded to a decrease in intracellular polyamine pools following the SBP-101 treatment. BENSpm, also a spermine analogue, is biochemically well-characterized and was used to compare the SBP-101 effects to previously described polyamine analogue effects. BENSpm decreased levels of all three individual polyamines: putrescine, spermidine, and spermine, in vitro. SBP-101 also decreased the intracellular levels of polyamines, but only decreased levels of two individual polyamines: putrescine and spermidine, in vitro. This is likely due to SBP-101’s more modest effect on SSAT activity. The upregulation of polyamine catabolism by the BENSpm treatment through the super-induction of SSAT reduces the overall spermine levels, whereas the SBP-101 treatment elicits only a modest induction of SSAT activity, resulting in limited spermine catabolism. The downregulation of the biosynthetic enzyme ODC by SBP-101 is more dramatic and more consistent across all of the cell lines than the upregulation of SSAT is, likely indicating that the primary method of polyamine depletion by SBP-101 is through the downregulation of polyamine biosynthesis. 

Our in vitro work demonstrated that SBP-101 was most effective in reducing cellular viability in ovarian cancer cells. Using the highly aggressive, immunosuppressive VDID8^+^ mouse ovarian cancer model, we investigated the potential of SBP-101 as an anti-tumor agent in vivo. A high-dose (24 mg/kg) SBP-101, administered Monday, Wednesday, and Friday on alternating weeks, produced a median survival of 65.5 days, a 42% increase in survival compared to untreated mice. This was accompanied by a delay in ascites onset, as well as an overall decrease in the ascites produced. It is important to note that there were some weight-related toxicities with this SBP-101 dosing schedule, with three animals being euthanized due to weight loss, not tumor burden. Additionally, that SBP-101 seen to accumulate in the livers and kidneys of treated animals suggests a potential depot effect. A subsequent study, treating with 6 mg/kg SBP-101 thrice per alternating week, demonstrated no weight-related toxicities and produced a 36% increase in the median survival time (62-days), comparable to the high dose SBP-101 treatment. The increased median survival was correlated with a decrease in the ascites polyamine levels, suggesting that SBP-101 is successful in modulating polyamine metabolism. The cells analyzed from the ascites fluid likely contained immune cells, tumor cells, and other cells from the tumor microenvironment. As the cells from the ascites were not sorted, the current data do not indicate which specific cell population(s) are undergoing changes to their polyamine metabolism in vivo; however, future experiments will determine the influences of SBP-101 on polyamines in immune cells and tumor cells, individually. 

The modulation of polyamine metabolism is a promising therapeutic option for the treatment of a variety of polyamine-dependent cancers. Polyamine analogues can be used to affect the activity of polyamine-related enzymes and deplete the intracellular polyamine pools on which neoplastic cells rely. SBP-101 is a promising spermine analogue currently being clinically evaluated in pancreatic cancer. Here we have shown that SBP-101 reduces cellular viability in vitro across numerous cancer types, likely due to a reduction in available intracellular polyamines. The reduction in intracellular polyamines is correlated with a strong downregulation of polyamine biosynthesis. SBP-101 reduces the polyamine content in cells found in the ascites fluid of an in vivo ovarian cancer mouse model. This correlates with a decrease in tumor burden and an increase in the life span of animals treated with SBP-101. Overall, our data suggest that SBP-101 is a viable modulator of polyamine metabolism and a potential therapeutic option for ovarian cancer in addition to pancreatic cancer. 

## 4. Materials and Methods

### 4.1. Cell Lines, Culture Conditions, and Reagents

Lung adenocarcinoma cell lines, A549 and H157, were maintained in RPMI containing 10% bovine calf serum (BCS) (Gemini Bio-Products, West Sacramento, CA, USA). Pancreatic adenocarcinoma lines, AsPC-1 and BxPC-3, were maintained in RPMI containing 10% fetal bovine serum (FBS) (Gemini Bio-Products, West Sacramento, CA, USA). Ovarian adenocarcinoma lines, CaOV-3 and SK-OV-3, were maintained in DMEM containing 10% FBS and McCoy’s 5a medium supplemented with 10% FBS, respectively. A549, H157, AsPC-1, BxPC-3, CaOV-3, and SK-OV-3 cell lines were all obtained from ATCC (Manassas, VA, USA). ID8 mouse ovarian surface epithelial cells (MOSE), overexpressing VEGF and β-Defensin (VDID8^+^), were maintained in RPMI supplemented with 10% FBS and obtained from Dr. Cynthia Zahnow [28,29]. The polyamine analogue BENSpm was synthesized as previously reported [30]. The novel polyamine analogue diethyl dihydroxyhomospermine (SBP-101) was obtained from Panbela Therapeutics, Inc. (Waconia, MN, USA) [23]. 

### 4.2. Cell Proliferation Assay and IC_50_ Determinations

Cells were seeded with a total cell count of 3 × 10^5^ cells per 25-cm^2^ flask and allowed to adhere overnight. Cells then received a fresh medium with increasing concentrations of either BENSpm or SBP-101. Following 96 or 120 h of drug exposure, cells were trypsinized, washed with PBS, and counted with an automated cell counter. Viable cells were determined by their ability to exclude trypan blue. IC_50_s were calculated using a non-linear regression equation for normalized data: Y = 100/(1 + X/IC_50_). 

### 4.3. Polyamine Enzyme Assays and Intracellular Polyamine Pool Determinations

Lysates from cells treated with either BENSpm or SBP-101 were used for ODC and SSAT enzyme activity assays using previously reported methods [31,32,33]. Perchloric acid-extracted lysates were labeled with dansyl chloride (Sigma-Aldrich, St. Louis, MO, USA) for detection using HPLC as previously described [34]. All enzymes’ activities and polyamine concentrations were quantified relative to total cellular protein in the lysate, as determined by a Bradford assay (Bio-Rad Laboratories, Hercules, CA, USA) [35]. 

### 4.4. Syngeneic Mouse Model

Female C57BL/6 wild-type mice (7–8 weeks old) were housed at the Johns Hopkins Sidney Kimmel Comprehensive Cancer Center Animal Resources Core and cared for in accordance with the policies set forth by the Johns Hopkins University Animal Care and Use Committee (Baltimore, MD, USA).

250,000 VEGF-β-Defensin ID8 (VDID8^+^) syngeneic mouse ovarian surface epithelial cells were injected intraperitoneally into C57BL/6 mice. Treatment of mice began three days following VDID8^+^ injection. Mice in the pilot study were treated with 24 mg/kg SBP-101 on Monday, Wednesday, and Friday, alternating weeks. Subsequent studies utilized two SBP-101 doses: 24 mg/kg once per alternating week and 6 mg/kg on Monday, Wednesday, and Friday, alternating weeks. All experimental arms included 10 mice, and all doses of SBP-101 were administered intraperitoneally. 

### 4.5. Mouse Tissue Processing and Polyamine Analysis

Ascites was collected from any animal showing greater than 20% weight gain (typically once or twice weekly following initial ascites formation). Processed ascites was passed through 45-μm filters and then incubated in ACK buffer (Thermo Fisher Scientific, Waltham, MA, USA) to lyse red blood cells. The remaining cells (both tumor and cells from the tumor microenvironment) were washed and lysates were prepared for polyamine analysis, as above. Polyamine levels were determined via HPLC as previously described [34]. 

Upon necropsy, livers and kidneys were removed from a subset of each mouse treatment group. The organs were homogenized in glycine buffer and polyamine levels were analyzed via HPLC as previously described [34]. 

### 4.6. Statistical Analysis

Ascites cell polyamine content was analyzed by the Shapiro–Wilk test for normality—all data sets passed the Shapiro–Wilk test (GraphPad Prism v. 9.3.1). Statistical analysis of ascites’ cell polyamine content was then completed using an unpaired parametric t-test. Animal survival curves were analyzed using a log-rank (Mantel-Cox) test. *p*-value indications are as follows:

* < 0.05; ** < 0.01; *** < 0.001; **** < 0.0001.

## Figures and Tables

**Table 1:** IC_50_ values following SBP-101 treatment in cancer cell lines.

Cell Line	IC_50_
A549	4.9 μΜ
H157 AsPC-1 BxPC-3 CaOV-3 Sk-OV-3	6.3 μM 4.4 μΜ 8.6 μM 1.3 μM 1.6 μΜ

**Table 2:** Intracellular analogue concentrations following treatment.

Cell Line	BENSpm (nmol/mg Protein)	SBP-101 (nmol/mg Protein)
A549	29.62	16.13
H157	26.87	31.21
AsPC-1	39.17	9.79
BxPC-3	29.13	19.11
CaOV-3	17.838	12.608
Sk-OV-3	24.573	12.199

## Data Availability

Data supporting reported results are contained within this article.

## Supplementary Materials

The following supporting information can be downloaded at: https://www.mdpi.com/article/10.3390/ijms23126798/s1.
