# Supplementary material for: Expanded Potential of the Polyamine Analogue SBP-101 (Diethyl Dihydroxyhomospermine) as a Modulator of Polyamine Metabolism and Cancer Therapeutic
Source: Int J Mol Sci. 2022 Jun 18;23(12):6798. doi: 10.3390/ijms23126798 (PMC9224330; doi:10.3390/ijms23126798)
Supplement: Supplementary file 1 [file ijms-23-06798-s001.zip › ijms-1774591-supplementary.pdf]

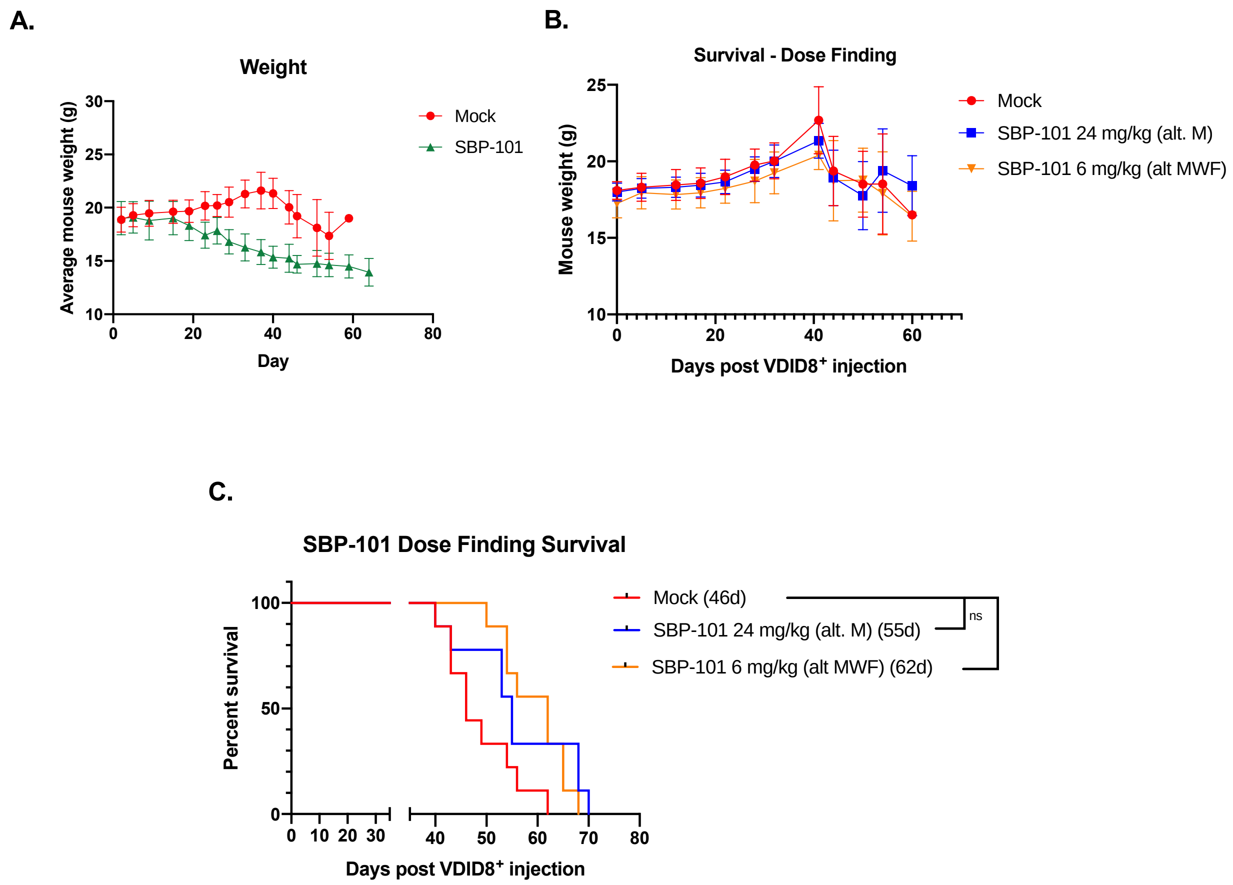

Supplementary Figure S1. Murine response to varying doses of SBP-101. Mice treated with 24 mg/kg SBP-101 3x per week on alternating weeks began to show weight loss around 22 days. SBP-101 treatment was stopped after 3 cycles (day 35 post VDID8^{+} injection) and weight subsequently stabilized **(A).** Mice treated with either 24 mg/kg SBP-101 once per alternating week or 6 mg/kg thrice per alternating week did not exhibit any observable weight loss **(B)**. The lower dose (6 mg/kg) of SBP-101 produced a median survival of 62 days ($p = 0.0067$), a similar survival benefit to that of the original 24 mg/kg **(C).**

Supplementary Table S1. SBP-101 deposits in the kidney and liver of treated mice. Kidneys and livers (from five control mice and five mice treated with 24 mg/kg three times per alternating week) were removed during necropsy and homogenized. The level of SBP-101, putrescine (PUT), spermidine (SPD), and spermine (SPM) was determined in the homogenized tissue samples by HPLC. The average content of each group is listed (all measured in nmol/mg protein). SBP-101 accumulated in the both the kidneys and the liver of treated mice, though the levels of SBP-101 were approximately 13-fold higher in the kidneys. SBP-101-treated mice have lower spermidine content in both the liver and kidney when compared to control mice.

Organ	Treatment	SBP-101 (nmol/mg protein)	PUT (nmol/mg protein)	SPD (nmol/mg protein)	SPM (nmol/mg protein)
Kidney	Control	0	2.87	19.33	17.73
Kidney	SBP-101	10.98	3.15	11.94	14.37
Liver	Control	0	3.59	69.75	23.94
Liver	SBP-101	1.01	2.13	26.97	21.75
